# Anti-Oxidant, Anti-Aging, and Anti-Melanogenic Properties of the Essential Oils from Two Varieties of *Alpinia zerumbet*

**DOI:** 10.3390/molecules200916723

**Published:** 2015-09-14

**Authors:** Pham Thi Be Tu, Shinkichi Tawata

**Affiliations:** 1 Department of Bioscience and Biotechnology, The United Graduate School of Agricultural Sciences, Kagoshima University, Korimoto 1-21-24, Kagoshima 890-0065, Japan; E-Mail: ptbetu@yahoo.com; 2 Department of Bioscience and Biotechnology, Faculty of Agriculture, University of the Ryukyus, Senbaru 1, Nishihara-cho, Okinawa 903-0129, Japan

**Keywords:** *Alpinia zerumbet*, essential oil, anti-aging, anti-oxidant, anti-melanogenic

## Abstract

Here, we investigated the anti-oxidant and anti-aging effects of essential oils (EOs) from the leaves of *Alpinia zerumbet* (*tairin* and *shima*) *in vitro* and anti-melanogenic effects in B16F10 melanoma cells. The anti-oxidant activities were performed with 2,2-diphenyl-1-picrylhydrazyl (DPPH); 2,2ʹ-azino-bis(3-ethylbenzothiazoline-6-sulfonic acid) diammonium salt (ABTS); nitric oxide; singlet oxygen; hydroxyl radical scavenging; and xanthine oxidase. The inhibitory activities against collagenase, elastase, hyaluronidase, and tyrosinase were employed for anti-aging. The anti-melanogenic was assessed in B16F10 melanoma cells by melanin synthesis and intracellular tyrosinase inhibitory activity. The volatile chemical composition of the essential oil was analyzed with gas chromatography-mass spectrometry (GC/MS). The EO was a complex mixture mainly consisting of monoterpenes and sesquiterpenes. The results revealed that *tairin* and *shima* EOs showed strong anti-oxidant activities against DPPH and nitric oxide, hydroxyl radical scavenging activity, and xanthine oxidase inhibition. Compared to *shima* EO; *tairin* EO exhibited strong anti-aging activity by inhibiting collagenase, tyrosinase, hyaluronidase, and elastase (IC_50_ = 11 ± 0.1; 25 ± 1.2; 83 ± 1.6; and 213 ± 2 μg/mL, respectively). Both EOs inhibited intracellular tyrosinase activity; thus, reducing melanin synthesis. These results suggest that *tairin* EO has better anti-oxidant/anti-aging activity than *shima* EO, but both are equally anti-melanogenic.

## 1. Introduction

Melanin, the major skin pigment, plays a crucial role in protecting human skin against ultraviolet (UV) radiation. However, overproduction and accumulation of melanin can result in pigmented patches and skin discolorations such as melasma, freckles, post-inflammatory melanoderma, and solar lentigo [1]. Melanin biosynthesis is regulated by melanogenesis enzymes, such as tyrosinase, which catalyzes two different reactions. In the initial step of melanin synthesis, l-tyrosine is hydroxylated to l-3,4-dihydroxyphenylalanine (l-DOPA, *O-*diphenol product). l-DOPA is oxidized further to the corresponding *O-*quinone. Thus, tyrosinase is an important regulator and the rate-limiting enzyme for melanogenesis [2]. Tyrosinase also regulates the browning of vegetables and foods. Therefore, melanin synthesis and tyrosinase inhibitors are used commonly in food processing and in cosmetics for melanin hyperpigmentation and skin whitening. Moreover, skin disorders are associated with increased dermal enzymatic activity, including collagenase, hyaluronidase, elastase, and tyrosinase [3,4,5,6]. Thus, inhibitors of these enzymes are increasingly important ingredients in cosmetics and medications to protect the skin against hyperpigmentation and skin aging.

UV radiation exposure induces formation of excess reactive oxygen species (ROS), which can interact with proteins, lipids, and DNA, and alter cellular functions, thus causing aging-related disorders or melanogenesis [7,8]. Recently, melanogenesis has been reported to produce hydrogen peroxide and other ROS in melanocytes [9]. This observation has increased interest in identifying natural antioxidants that inhibit skin aging and melanogenesis.

*Alpinia zerumbet* (Pers.) Burtt and Smith (alpinia), a member of the *Zingiberaceae* family, is a perennial plant that is distributed widely in subtropical and tropical regions and is used in folk medicine. In Okinawa, there are two varieties of alpinia, *tairin* (*Alpinia zerumbet* (Pers.) B. L. Burtt & R. M. Sm. var. excelsa Funak & T. Y. Ito) and *shima* (*Alpinia zerumbet* (Pers.) B. L. Burtt & R. M. Sm.). *Tairin* is a taller plant with long stems, whereas s*hima* is shorter with a bushy structure. The leaves of both plants are used in the preparation of a traditional food, *mu-chi*, and are thought to prevent common cold [10].

We previously demonstrated that alpinia leaves have anti-oxidant and anti-microbial activities [11,12]. We also reported that alpinia can inhibit advanced glycation end products, HIV-1 integrase, and neuraminidase (NA) [13,14]. Essential oils (EO) from alpinia also have been reported to have various biological activities such as a myorelaxant and anti-spasmodic agent in the rat ileum [15], anti-nociceptive in mice [16], and anti-hypertensive and cardiovascular disease preventative in rats [17]. In addition, alpinia EOs are associated with anti-microbial and larvicidal activity [18,19].

In this study, we examined the anti-oxidant and anti-aging activity of EOs isolated from the leaves of two varieties of alpinia. Furthermore, we evaluated the inhibitory activity of these samples against melanin biosynthesis in B16F10 melanoma cells.

## 2. Results and Discussion

### 2.1. Chemical Compositions of Tairin and Shima EOs

The major compounds in the *tairin* EO were γ-terpinene (14.5%), cineole (13.8%), *p*-cymene (13.5%), sabinene (12.5%), terpinen-4-ol (11.9%), caryophyllene oxide (4.96%), methyl cinnamate (4.24%), caryophyllene (2.4%), and γ*-*terpineol (1.28%). Whereas *shima* EO contained the main components of cineole (37.8%), β-linalool (17.1%), caryophyllene oxide (10.4%), methyl cinnamate (6.34%), benzylacetone (4.21%), and α*-*terpineol (3.36%). The chemical components of the essential oil from two varieties of alpinia leaf were presented in Table 1.

**Table 1:** Chemical components of the EO from two varieties of alpinia leaf.

No.	Compound	Retention Index	Peak Area (%)	
			*Tairin*	*Shima*
1	*m*-Cumenol	917	0.16	0.15
2	α*-*Thujene	929	4.12	-
3	α*-*Pinene	934	2.02	-
4	Norborndadiene	943	0.08	-
5	Camphene	947	0.22	-
6	Benzaldehyde	963	-	1.59
7	Sabinene	974	12.5	-
8	β*-*Pinene	976	3.15	-
9	Myrcene	989	0.69	-
10	α*-*Phellandrene	1002	0.31	-
11	*p-*Cymene	1024	13.5	-
12	1,8-Cineole	1031	13.8	37.8
13	γ*-*Terpinene	1059	14.5	-
14	cis*-*β*-*Terpineol	1068	0.55	-
15	β*-*Linalool	1100	0.50	17.1
16	2,5-Norbornadiene	1111	-	0.71
17	cis*-p-*Menth*-*2*-*en*-*1*-*ol	1121	0.59	-
18	Borneol	1154	0.31	0.11
19	Terpinen-4-ol	1173	11.9	-
20	γ-Terpineol	1193	1.28	3.36
21	*trans-p-*Menth*-*1*-*en*-*3*-*ol	1206	0.42	-
22	Methyl cinnamate	1233	4.24	6.32
23	Benzylacetone	1237	0.06	4.21
24	Piperitone	1248	0.03	0.1
25	Bornyl acetate	1280	0.37	-
26	Cumic alcohol	1287	0.18	-
27	2-*tert*-Butylphenyl pivalate	1299	-	1.23
28	Ethyl-3-hydroxy-3-methylbutanote	1302	-	0.09
29	Isopiperitenon	1306	-	0.18
30	Thymol	1317	0.05	-
31	2-Hydroxy-3,5-dimethylcyclopent-2-en-1-one	1319	-	0.13
32	*p-*Menth*-*1,4*-*dien*-*7*-*ol	1324	0.07	-
33	Cymen-8-ol	1354	0.26	0.29
34	Carvacrol	1379	1.61	1.89
35	Caryophyllene	1411	2.40	-
36	2,6-Diethylnitrosobenzene	1426	-	0.69
37	α*-trans-*Bergamoene	1428	0.09	-
38	Aristole*-*9*-*ene	1434	0.15	-
39	α*-*Humulene	1446	0.38	-
40	*p*-Cymen-7-ol	1447	1.66	1.77
41	γ*-*Cadinene	1505	0.42	-
42	α*-*Bulnesene	1514	0.25	-
43	Nerolidol	1559	0.38	-
44	Caryophyllene oxide	1680	4.96	10.4
45	α*-*Zingiberene	1701	0.02	-
	Total		98.18	88.12

### 2.2. Anti-Oxidant Activities

#### 2.2.1. DPPH and ABTS Radical Scavenging Activities

The scavenging activity of *tairin* and *shima* EOs was determined using 2,2-diphenyl-1-picrylhydrazyl (DPPH) free radicals (Table 2). The results indicate that *tairin* EO exhibited higher antioxidant activity (IC_50_ = 5.7 ± 0.6 μg/mL) than *shima* EO (IC_50_ = 126 ± 1.1 μg/mL). The DPPH radical scavenging activity of *tairin* EO was also higher than that of the positive control, tert-butyl hydroxytoluene (BHT) (IC_50_ = 29 ± 0.6 μg/mL).

The scavenging capacity of *tairin* and *shima* EOs was also assessed using the 2,2ʹ-azino-bis(3-ethylbenzothiazoline-6-sulfonic acid) diammonium salt (ABTS) radical cation. The data indicate that the ABTS scavenging activity differed considerably between the EOs. The ABTS radical scavenging activity of *tairin* EO (IC_50_ = 260 ± 1.8 μg/mL) was greater than that of *shima* EO (IC_50_ = 337 ± 2.1 μg/mL) (Table 2).

**Table 2:** Antioxidant activity of *tairin* and *shima* essential oils (EOs).

Sample	Anti-Oxidant Activities (IC_50_, μg/mL)				
	DPPH	ABTS	Nitric Oxide	Singlet Oxygen	Hydroxyl Radical Scavenging
*Tairin*	5.7 ± 0.6 ^a^	260.5 ± 1.8 ^b^	66.3 ± 1.6 ^b^	2145 ± 2.8 ^c^	69.2 ± 2.1 ^a^
*Shima*	126.3 ± 1.1 ^c^	337.1 ± 2.1 ^c^	704.2 ± 1.4 ^c^	503 ± 1.7 ^b^	74.0 ± 1.2 ^b^
BHT	29.1 ± 0.6 ^b^	45.7 ± 1.7 ^a^	-	-	-
Ascorbic acid	-	-	14.4 ± 0.8 ^a^	-	72.3 ± 1.2 ^b^
Rutin	-	-	-	54.0 ± 0.2 ^a^	-

The values represent the mean ± standard error (*n =* 3). Letters with different superscripts indicate samples that are significant different (*p* < 0.05) to each other. IC, inhibitory concentration; DPPH, 2,2-diphenyl-1-picrylhydrazyl; ABTS, 2,2ʹ-azino-bis(3-ethylbenzothiazoline-6-sulfonic acid) diammonium salt; BHT, tert-butyl hydroxytoluene. (-): not detected.

#### 2.2.2. Singlet Oxygen Scavenging (^1^O_2_) Activity

^1^O_2_ is a high-energy state molecular oxygen species. It is one of the most active intermediates involved in chemical and biochemical reactions. The singlet oxygen scavenging activity of *tairin* and *shima* EOs was compared with that of rutin (Table 2). *Shima* EO showed significantly (*p* < 0.05) higher ^1^O_2_ scavenging activity (IC_50_ = 503 ± 1.7 μg/mL) than *tairin* EO (IC_50_ = 2145 ± 2.8 μg/mL).

#### 2.2.3. Hydroxyl and Nitric Oxide Radical Scavenging Activities

The hydroxyl radical scavenging activity of *tairin* and *shima* EOs was determined as the percent inhibition of hydroxyl radical formation in the Fenton reaction. The scavenging activity of *tairin* and *shima* EOs was compared with that of ascorbic acid. *Tairin* EO (IC_50_ = 69 ± 2.0 μg/mL) had significantly higher (*p* < 0.05) hydroxyl radical scavenging activity and was more potent than ascorbic acid (IC_50_ = 72 ± 1.2 μg/mL), whereas *shima* EO had an IC_50_ = 74 ± 1.2 μg/mL (Table 2).

Nitric oxide, generated from sodium nitroprusside in aqueous solutions, interacts with oxygen to produce nitrite ions, which can be measured by the Griess reaction. The EOs from *tairin* and *shima* showed scavenging activity between 66 and 704 μg/mL. The percent inhibition increased with increasing concentrations of EO. *Tairin* EO inhibited nitric oxide formation better (IC_50_ = 66 ± 1.6 μg/mL) than s*hima* EO (IC_50_ = 704 ± 1.5 μg/mL) (Table 2). However, ascorbic acid inhibited nitric oxide better than both EOs, with an IC_50_ value of 14 ± 0.8 μg/mL.

### 2.3. Anti-Aging Activities

#### 2.3.1. Collagenase and Elastase Assay

**Figure 1:**
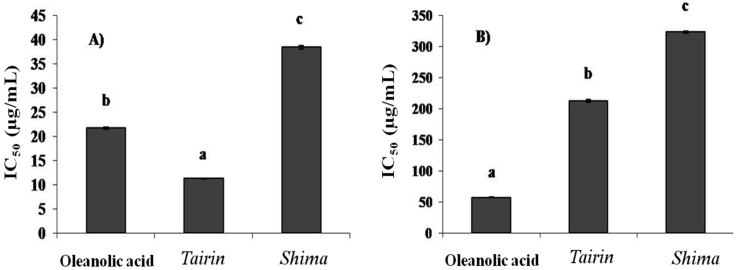
*Tairin* and *shima* essential oil (EO)-mediated collagenase (**A**) and elastase (**B**) inhibition. Values shown represent the mean ± standard error from three independent experiments. One-way ANOVA was used for the comparison of multiple group means, followed by the Duncan test (*p* < 0.05). Letters with different superscripts indicate samples that are significantly different (*p* < 0.05) from each other. IC, inhibitory concentration.

As shown in Figure 1A,B, the EOs from *shima* and *tairin* inhibited collagenase and elastase activities. Both *tairin* and *shima* EOs showed strong collagenase inhibitory activities (IC_50_ = 11 ± 0.1 μg/mL and 38 ± 0.4 μg/mL, respectively). Further, *tairin* EO inhibited collagenase better than the positive control, oleanolic acid (IC_50_ = 21 ± 0.2 μg/mL). A similar profile was observed when evaluating elastase activity. *Tairin* EO showed higher inhibitory activity (IC_50_ = 212 ± 2.1 μg/mL) than *shima* EO (IC_50_ = 322 ± 0.5 μg/mL).

#### 2.3.2. Tyrosinase and Hyaluronidase Inhibition Assay

**Figure 2:**
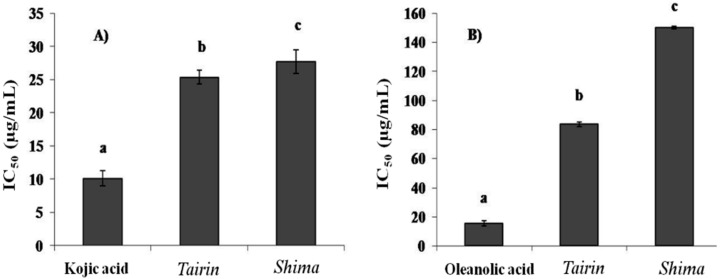
*Tairin* and *shima* essential oil (EO)-mediated tyrosinase (**A**) and hyaluronidase (**B**) inhibition. Values shown represent the mean ± standard error from three independent experiments. One-way ANOVA was used for the comparison of multiple group means, followed by the Duncan test (*p* < 0.05). Letters with different superscripts indicate samples that are significantly different (*p* < 0.05) from each other. IC, inhibitory concentration.

Tyrosinase is a multifunctional, glycosylated, copper-containing oxidase that catalyzes the first two steps of mammalian melanogenesis and is responsible for enzymatic browning reactions in damaged fruits during post-harvest handling and processing. In this study, we evaluated ability of EOs from *tairin* and *shima* to inhibit tyrosinase. *Tairin* EO (IC_50_ = 25 ± 1.2 μg/mL) inhibited tyrosinase better than *shima* EO (IC_50_ = 28 ± 1.5 μg/mL). However, kojic acid inhibited tyrosinase better than both EOs (IC_50_ = 10 ± 0.7 μg/mL) (Figure 2A).

The hyaluronidases are a group of enzymes distributed throughout the animal kingdom. In recent years, hyaluronidase has received attention owing to its ability to regulate hyaluronan metabolism. Hyaluronidase inhibitors are potent regulators that maintain hyaluronidase homeostasis and may serve as anti-inflammatory agents. Our results indicate that *tairin* EO is a stronger inhibitor (IC_50_ = 83 ± 1.6 μg/mL) of hyaluronidase than *shima* EO (IC_50_ = 150 ± 0.8 μg/mL) (Figure 2B).

#### 2.3.3. Xanthine Oxidase Inhibition Assay

**Figure 3:**
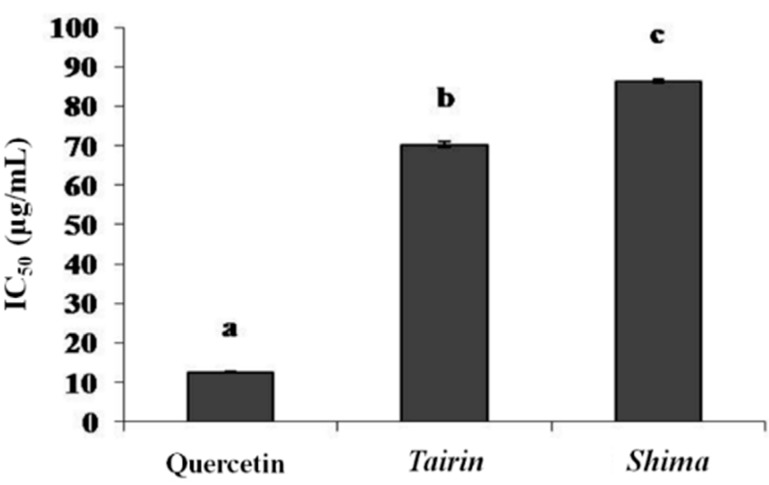
Inhibition of xanthine oxidase activity by *tairin* and *shima* essential oils (EOs). Values shown represent the mean ± standard error from three independent experiments. One-way ANOVA was used for the comparison of multiple group means, followed by the Duncan test (*p* < 0.05). Letters with different superscripts indicate samples that are significant different (*p* < 0.05) from each other. IC, inhibitory concentration.

Xanthine oxidase is a flavoprotein that catalyzes the oxidation of hypoxanthine to xanthine and generates superoxide and uric acid. Xanthine oxidase inhibitors may be useful for the treatment of skin aging and gout, which is caused by the generation of uric acid and the superoxide anion radical. Our results indicate that *tairin* EO significantly inhibits xanthine oxidase (IC_50_ = 70 ± 0.6 μg/mL) and is a stronger inhibitor than *shima* EO (IC_50_ = 86 ± 0.4 μg/mL) (Figure 3).

### 2.4. Anti-Melanogenic Effects of Tairin and Shima EOs

#### 2.4.1. Cell Viability

**Figure 4:**
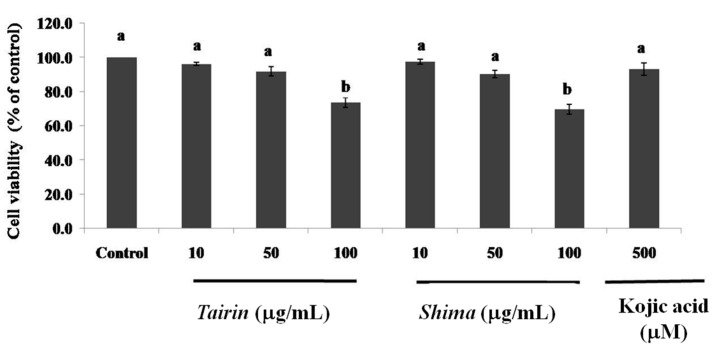
Effect of *tairin* and *shima* essenetial oils (EOs) on B16F10 melanoma cell viability. B16F10 cells were treated with various concentrations of EO and incubated for 48 h. Values shown represent the mean ± standard error for three independent experiments. One-way ANOVA was used for the comparison of multiple group means, followed by the Duncan test (*p* < 0.05). Letters with different superscripts indicate samples that are significantly different (*p* < 0.05) from each other.

To analyze the effects of *tairin* and *shima* EOs on cell viability, B16F10 melanoma cells were treated with varying concentrations of EO for 48 h. As shown in Figure 4, the *tairin* and *shima* EOs did not affect the cell viability up to 50 μg/mL; however, treatment with 100 μg/mL caused a 26% ± 3% and 30% ± 3% decrease in cell viability, respectively. Thus, the *tairin* and *shima* EOs were used at concentrations up to 50 μg/mL to evaluate their effects on melanin content and intracellular tyrosinase activity.

#### 2.4.2. Effects of *Tairin* and *Shima* EOs on Melanin Content

**Figure 5:**
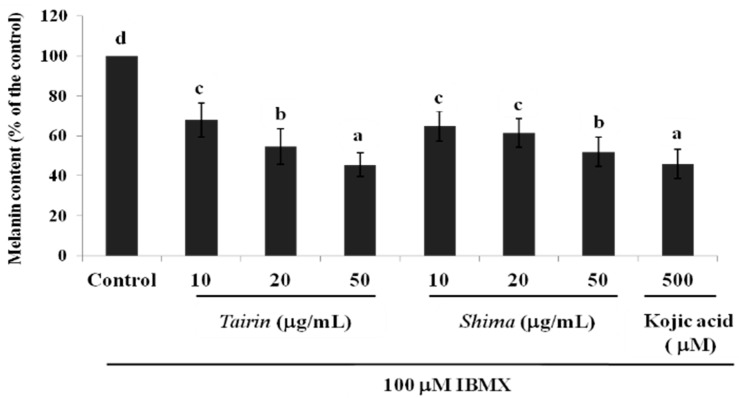
Effect of *tairin* and *shima* essential oils (EOs) on melanin production in B16F10 melanoma cells. B16F10 cells were treated with various concentrations of EO and incubated for 48 h. The melanin content of B16F10 melanoma cells was measured with a microplate reader at 490 nm. Values shown represent the mean ± standard error for three independent experiments. A one-way ANOVA was used for the comparison of multiple group means, followed by the Duncan test (*p* < 0.05). Letters with different superscripts indicate samples that are significant different (*p* < 0.05) from each other.

To determine the anti-melanogenic activity of *tairin* and *shima* EOs, their effect on melanin content was evaluated in B16F10 melanoma cells. The B16F10 melanoma cells were treated with varying concentrations of *tairin* and *shima* EOs and then stimulated with IBMX for 48 h. As shown in Figure 5, *tairin* and *shima* EOs dose-dependently inhibited melanin content. At 50 μg/mL, melanin synthesis was inhibited 55% ± 6% and 48% ± 7%, respectively. Kojic acid, the positive control, inhibited melanin content 54% ± 7%. These results demonstrated that both *tairin* and *shima* EOs decreased the melanin content without any effect on the cell viability.

#### 2.4.3. Inhibition of the Intracellular Tyrosinase Activity by *Tairin* and *Shima* EOs

**Figure 6:**
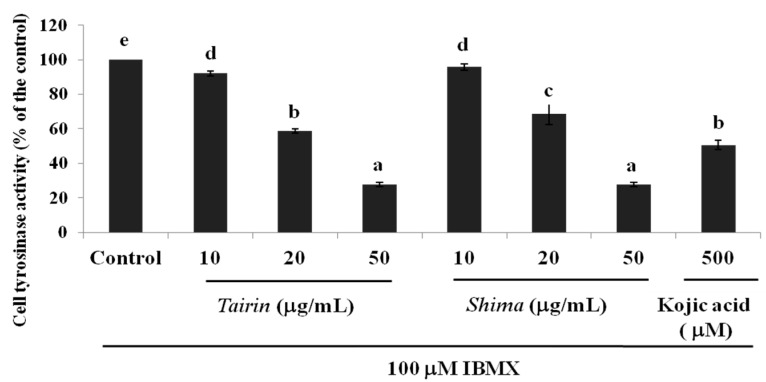
Effect of *tairin* and *shima* essential oils (EOs) on intracellular tyrosinase activity in B16F10 melanoma cells. B16F10 cells were treated with various concentrations of EO and incubated for 48 h. The intracellular tyrosinase activity of B16F10 melanoma cells was measured with a microplate reader at 490 nm. Values shown represent the mean ± standard error for three independent experiments. One-way ANOVA was used for the comparison of multiple group means, followed by the Duncan test (*p* < 0.05). Letters with different superscripts indicate samples that are significant different (*p* < 0.05) from each other.

To evaluate whether *tairin* and *shima* EOs inhibit the intracellular tyrosinase activity, the B16F10 melanoma cells were treated with varying concentrations of the EOs for 48 h, followed by incubation with l-DOPA. Our data indicate that, following treatment with 20 μg/mL *tairin* and *shima* EOs, tyrosinase activity was inhibited by 41% ± 1% and 31% ± 6%, respectively. At a concentration of 50 μg/mL, both *tairin* EO (72% ± 1%) and *shima* EO (71% ± 1%) inhibited tyrosinase activity more potently than that by the positive control kojic acid (49% ± 3%) (Figure 6). These results indicate that *tairin* and *shima* EOs could be applicable in cosmetics as skin-whitening agents for the treatment of pigmentation disturbances.

### 2.5. Discussion

The safety of natural products used in drugs, health foods, and cosmetic ingredients is a major concern. Several studies have explored the use of extracts from the leaves of *Aesculus hippocastanum* and Bunge [20,21], the gel of *Aloe vera* [22], the oil of *Camellia japonica* L. [23], and the rhizomes of *Zingiber officinale* L. [24] to prevent skin abnormalities and hyperpigmentation. In this study, we first determined the free radical scavenging activities of *tairin* and *shima* EOs. Our study demonstrated that *tairin* had stronger anti-oxidant activity than *shima*, with the exception of singlet oxygen scavenging activity. The GC/MS analysis showed *tairin* and *shima* EOs contain the major components of monoterpenes and sesquiterpenes. Previous studies have been reported that monoterpenes, such as terpinen-4-ol, 1,8-cineol, and γ-terpinene, showed anti-oxidant activities [11,12,25,26]. The strong anti-oxidant activity from *tairin* and *shima* EOs may be attributed from these components. It is known that UV radiation induces free radical formation in the skin, which is linked directly to the onset of skin photodamage and biological damage. Thus, our results suggest that *tairin* and *shima* EOs may be useful anti-oxidant sources to prevent UV-induced damage.

Next, we highlighted the anti-aging activity of EOs from *tairin* and *shima* through collagenase, elastase, hyaluronidase, and tyrosinase enzyme inhibition assays, and compared these results with those of oleanolic acid, which is a skin-protective pentacyclic triterpene. UV light stimulates collagenase expression, which causes matrix protein degradation and, subsequently, skin photoaging [27]. Elastase and hyaluronidase are proteolytic enzymes in the dermis that are responsible for the degradation of elastin and hyaluronan, respectively, in the extracellular matrix. Loss of elastin is a major part of what cause visible signs of aging (wrinkles, sagging) in the skin. Additionally, in human melanocytes, melanin synthesis is regulated by tyrosinase, leading to skin diseases. Our results indicate that *tairin* EO was a stronger tyrosinase, elastase, and hyaluronidase inhibitor than *shima* EO.

To our knowledge, the inhibitory effect of *tairin* and *shima* EOs on melanogenesis is reported here for the first time. IBMX is a well-known stimulator of melanogenesis, leading to tyrosinase synthesis and increased melanin levels [28]. We found that the EOs decreased melanin content in melanoma cells more effectively than the commercial melanin synthesis inhibitor (kojic acid). In order to understand better the potential use of *tairin* and *shima* EOs to reduce melanogenesis, we also evaluated the inhibition of intracellular tyrosinase activity. The present study indicated that *tairin* and *shima* EOs strongly inhibited intracellular tyrosinase activity. Thus, these EOs may be good candidates for use in skin-whitening materials.

In addition, we previously found a few RAC/CDC42-activated kinase 1 (PAK1)-blocking compounds in extracts of alpinia [29] and recently have revealed that PAK1 is essential for melanogenesis as well as the aging process and oncogenesis [30,31]. Thus, it is most likely that the anti-aging and anti-melanogenic effects of alpinia EOs could be partly attributed to their PAK1-blocking activity.

## 3. Experimental Section

### 3.1. General

2,2-diphenyl-1-picrylhydrazyl (DPPH), tert-butyl hydroxytoluene (BHT), kojic acid, 2,2ʹ-azino-bis(3-ethylbenzothiazoline-6-sulfonic acid) diammonium salt (ABTS), quercetin, l-tyrosine, hydrogen peroxide, sodium hypochloride, histidine, ion (III) chloride, potassium dihydrogen phosphate, hydrochloric acid, potassium hydroxide, Dulbecco’s modified Eagle medium (DMEM), fetal bovine serum (FBS), Triton-X, and bovine serum albumin (BSA) were purchased from Wako Pure Chemical Industries, Ltd. (Osaka, Japan). *N*-suc-(Ala)3-nitroanilide (SANA), *N*-[3-(2-furyl) acryloyl]-Leu-Gly-Pro-Ala (FALGPA), collagenase, elastase, hyaluronidase, tyrosinase, hyaluronic acid, oleanolic acid, sodium nitroprusside (SNP), *N*,*N*-dimethyl-4-nitrosoaniline (RNO), sulfanilic acid, trichloroacetic acid, thiobarbituric acid, ascorbic acid, 2-deoxy-2 ribose, l-tyrosine, l-DOPA, 3-(4,5-dimetylyhiazol-2-yl)-2,5-diphenyltetrazolium bromide (MTT), dimethyl sulfoxide (DMSO), and *N*-(1-naphthy) ethyenediamine dihydrochloride (NED) were obtained from Sigma-Aldrich, Inc. (St. Louis, MO, USA). Xanthine was purchased from Kanto Chemical Co., Inc. (Tokyo, Japan). Xanthine oxidase was purchased from Toyobo Co., Ltd. (Osaka, Japan). Ethylenediaminetetraacetic acid (EDTA) was obtained from Kanto Chemical Co, Inc. (Tokyo, Japan). B16F10 melanoma cells were purchased from American Type Culture Collection (Rockville, MD, USA).

### 3.2. Extraction of EO from Alpinia Leaf

The EOs from fresh *tairin* and *shima* leaves (500 g) were isolated by steam distillation for 4 h. The distillate was extracted with diethyl ether, and the solvent was removed carefully under vacuum at 35 °C. The obtained EOs were dissolved in methanol and dimethyl sulfoxide (DMSO) and kept under refrigeration until use.

### 3.3. Gas Chromatography-Mass Spectrometry (GC-MS) Analysis

The compounds of EO were identified using DB-5MS fused silica capillary column (30 m × 0.25 mm i.d., 0.25 μm; Agilent Technologies, J & W Scientific Products, Folsom, CA, USA). The carrier gas was helium and the GC oven temperature program was as follows: 80 °C hold for 1 min, increase 10 °C/min to 220 °C, followed by increase to 330 °C at 20 °C/min and hold for 6 min. The injector and detector temperatures were set at 250 °C and 280 °C, respectively, and the injection volume was 1.0 μL in the splitless mode. Mass spectra were scanned form *m*/*z* 50–600 amu and the electron impact ionization energy was 70 eV. Quantitative determinations of EO components were done based on the peak area measurements. Retention indices were determined relative to the retention times of a series of *n*-alkane standards (C7 to C31, Restek Corporation, PA, USA), and compared with published values [11,12].

### 3.4. Anti-Oxidant Activity

#### 3.4.1. DPPH Radical Scavenging Assay

The DPPH radical scavenging assay was performed according to Boskou *et al.* [32]. DPPH (40 μL) and sodium acetate buffer (80 μL, 0.1 M, pH 5.5) were added to samples (80 μL) containing a range of EO concentrations (10, 100, and 250 μg/mL). The mixture was incubated at room temperature in the dark for 30 min. The absorbance was measured at 517 nm using a microplate spectrophotometer (Bio-Rad Laboratories, Inc., Hercules, CA, USA). BHT was used as positive control.

#### 3.4.2. ABTS Radical Scavenging Assay

The ABTS radical scavenging assay was carried out using a method previously described by Re *et al.* [33]. The working solution was prepared by mixing 7 mM ABTS and 2 mM K_2_S_2_O_8_ in equal quantities and allowing them to react in the dark overnight (more than 16 h) at room temperature. The solution was diluted with water to obtain an absorbance of 0.70 ± 0.03 units at 734 nm. The working ABTS solution (200 μL) was mixed with 20 μL of the samples containing varying EO concentrations (10, 100, and 250 μg/mL), and the absorbance was measured at 734 nm after 6 min of incubation at room temperature. BHT was used as a positive control.

#### 3.4.3. Hydroxyl Radical Scavenging (^•^OH) Assay

The hydroxyl radical scavenging assay was performed as described by Ozyurek *et al.* [34]. The reaction mixture for the deoxyribose assay contained the following reagents in a final volume of 1 mL: 200 μL of KH_2_PO_4_-KOH (100 mM), 200 μL of deoxyribose (15 mM), 200 μL of FeCl_3_ (500 μM), 100 μL of EDTA (1 mM), 100 μL of ascorbic acid (1 mM), 100 μL of H_2_O_2_ (10 mM), and 100 μL of sample. The reaction mixtures were incubated at 37 °C for 1 h. At the end of the incubation period, 1 mL of 2.8% (*w*/*v*) trichloroacetic acid (TCA) was added to each mixture, followed by the addition of 1 mL of 1% (*w*/*v*) thiobarbituric acid (TBA). The solutions were heated in a water bath at 90 °C for 15 min until a pink color developed, and the absorbance of the resulting solution was measured at 532 nm. Ascorbic acid was used as a positive control.

#### 3.4.4. Singlet Oxygen Scavenging (^1^O_2_) Assay

The production of singlet oxygen was determined by monitoring *p*-nitrosodimethylaniline (RNO) bleaching using a spectrophotometric method previously reported by Chakraborty and Tripathy [35]. Singlet oxygen was generated by reacting NaOCl and H_2_O_2_, and the bleaching of RNO was measured. The reaction mixture contained 45 mM phosphate buffer (pH 7.1), 50 mM NaOCl, 50 mM H_2_O_2_, 50 mM histidine, 10 μM RNO, and the test sample in a final volume of 2 mL. The reaction was incubated at 30 °C for 40 min, and the decrease in RNO absorbance was measured at 440 nm. Rutin was used as a positive control.

#### 3.4.5. Nitric Oxide Scavenging Assay

Nitric oxide radical scavenging activity was estimated using the Griess Illosvoy reaction [36]. The reaction mixture (300 μL), containing 10 mM SNP (200 μL), phosphate-buffered saline (50 μL), and the test sample (50 μL), was incubated at 25 °C for 150 min. After incubation, 50 μL of the reaction mixture was mixed with 100 μL of sulfanilic acid reagent (0.33% in 20% glacial acetic acid) and allowed to stand for 5 min for complete diazotization. NED (100 μL) was added, and the mixture was incubated at 25 °C for 30 min, until a pink chromophore could be observed in diffused light. The absorbances of these solutions were measured at 540 nm against the corresponding blank solutions. Ascorbic acid was used as the positive control, while methanol was used as the control for calculation.

The % scavenging capacities of DPPH, ABTS, hydroxyl radical, singlet oxygen, and nitric oxide were calculated using the formula:

% scavenging capacity = [(A_control_ − A_sample_)/A_control_] × 100 (1)

where A_control_ is the absorbance of the control without the test sample, and A_sample_ is the absorbance of the sample.

All experiments were carried out in triplicate. The % radical scavenging activity was plotted against the corresponding extract concentration to obtain the IC_50_ value.

### 3.5. Enzymatic Activities

#### 3.5.1. Collagenase Inhibition Assay

The collagenase inhibition assay was performed by measuring FALGPA hydrolysis using a previously described method [37]. The assay was performed in 50 mM tricine buffer, containing 400 mM NaCl and 10 mM CaCl_2_ (pH 7.5). *Clostridium histolyticum* collagenase (ChC) and the synthetic substrate, *N*-(3-[2-Furyl]acryloyl)-Leu-Gly-Pro-Ala (FALGPA) were dissolved in the tricine buffer for use at initial concentrations of 0.8 U/mL and 2 mM, respectively. The EOs was incubated with the enzyme in the buffer for 15 min before adding the substrate to start the reaction. The final reaction mixture (150 μL total volume) contained tricine buffer, 0.8 mM FALGPA, 0.1 U ChC, and 25 μg EO samples. Water and methanol were used as negative controls, while oleanolic acid was used as a positive control. After incubating with the substrate for 20 min, collagenase activity was measured at 340 nm.

#### 3.5.2. Elastase Inhibition Assay

Elastase inhibition was assayed using the method of Kransoe *et al.* [38] with slight modifications. Inhibition was determined by measuring the intensity of the solution color following elastase-mediated SANA cleavage. Briefly, 1 mM SANA was prepared in 0.1 M Tris-HCl buffer (pH 8.0). The solution (200 μL) was added to a stock sample solution (20 μL). The solutions were vortexed and preincubated for 10 min at 25 °C, and then 20 μL of elastase from porcine pancreas (0.03 U/mL) was added. After vortexing, each solution was placed in a water bath at 25 °C for 10 min. The absorbance was measured at 410 nm. Negative controls were performed with water and methanol, while oleanolic acid was used as a positive control.

#### 3.5.3. Hyaluronidase Inhibition Assay

Inhibition of hyaluronidase was determined as previously described [39]. A 5 μL test sample was pre-incubated with bovine hyaluronidase (1.50 U) in 100 μL of solution containing 20 mM (pH 7.0) sodium phosphate buffer, 77 mM sodium chloride, and 0.01% bovine serum albumin (BSA) for 10 min at 37 °C. The assay was initiated by adding 100 μL of hyaluronic acid sodium salt from rooster comb (0.03% in 300 mM sodium phosphate, pH 5.35) to the incubation mixture, followed by incubation for 45 min at 37 °C. Undigested hyaluronic acid was precipitated with acid albumin solution (1 mL), composed of 0.1% BSA in 24 mM sodium acetate and 79 mM acetic acid (pH 3.75). The solution was allowed to stand at room temperature for 10 min, and the absorbance was measured at 600 nm. Oleanolic acid was used as the positive control.

The percent collagenase, elastase, and hyaluronidase inhibition were calculated by:

Enzyme inhibition activity (%) = (1 − B/A) × 100
(2)

where A is the enzyme activity without sample, and B is the activity in the presence of the sample.

#### 3.5.4. Tyrosinase Inhibition Assay

Tyrosinase inhibition was determined by measuring DOPA chrome formation as previously described [40]. In brief, EO samples were dissolved in solvent to varying concentrations (10, 100, and 250 μg/mL). Samples were prepared in a 96-well plate, and the components were added as follows: phosphate buffer (120 μL, 20 mM, pH 6.8), 20 μL sample, and 20 μL mushroom tyrosinase (500 U/mL in 20 mM phosphate buffer). After incubation at 25 °C for 15 min, the reaction was initiated by adding 20 μL l-tyrosine solution (0.85 mM) to each well and incubating at room temperature for 10 min. The enzyme activity was determined by measuring the absorbance at 470 nm. Kojic acid was used as the positive control. The percent tyrosinase inhibition was calculated as follows:

Tyrosinase inhibition (%) = [(A − B) − (C − D)]/(A − B) × 100
(3)

where A is the absorbance of the control with the enzyme, B is the absorbance of the control without the enzyme, C is the absorbance of the test sample with the enzyme, and D is the absorbance of the test sample without the enzyme.

#### 3.5.5. Xanthine Oxidase Inhibition Assay

Xanthine oxidase activity was determined by measuring the formation of uric acid from xanthine [41]. Phosphate buffer (300 μL, 50 mM, pH 7.4) was used in this assay. Varying concentrations of EO (100 μL) were mixed with a solution containing xanthine oxidase (100 μL, 0.2 U/mL) and water (100 μL). Samples were then incubated at 37 °C for 15 min. Xanthine (200 μL, 0.15 mM) was then added to each sample and further incubated at 37 °C for 30 min. The reaction was stopped by adding HCl (200 μL, 0.5 M). The production of uric acid was determined by measuring the absorbance at 295 nm. The buffer was used as a blank, and the control was a solution containing xanthine and xanthine oxidase.

The percent xanthine oxidase inhibition was calculated by:

Enzyme inhibition activity (%) = (1 − B/A) × 100
(4)

where A is the enzyme activity without sample, and B is the activity in the presence of the sample.

### 3.6. Effects of Tairin and Shima EO on Melanin Biosynthesis in B16F10 Melanoma Cells

#### 3.6.1. Cell Culture

B16F10 melanoma cells were cultured in DMEM supplemented with 10% heat-inactivated FBS and 1% penicillin/streptomycin (10,000 U/mL and 100 μg/mL) at 37 °C in a humidified atmosphere containing 5% CO_2_.

#### 3.6.2. Cell Viability

Cell viability was determined after EO treatment using an MTT assay, as described by Campos *et al.* [42]. Briefly, B16F10 cells were plated at a density of 7 × 10^3^ cells/well in a 96-well plate. After 48 h of culture, cells were exposed to varying concentrations of *tairin* and *shima* EOs (10, 50, and 100 μg/mL) or 500 μM kojic acid and incubated for an additional 48 h at 37 °C. Following incubation, the medium was removed, and the cells were washed twice with phosphate buffer and incubated with MTT solution (0.5 mg/mL) for 3 h at 37 °C. The medium was discarded, and 200 μL of ethanol was added. The absorbance of each well was measured at 570 nm using a microplate spectrophotometer (Bio-Rad Laboratories, Inc., Hercules, CA, USA).

#### 3.6.3. Measurement of Melanin Content

Melanin content was determined as described by Yoon *et al.* [43]. In brief, B16F10 cells were plated at a density of 7 × 10^3^ cells/well in a 96-well plate. After 48 h of culture, cells were exposed to varying concentrations of *tairin* and *shima* EO (10, 20, and 50 μg/mL) or 500 μM kojic acid. After 1 h, 100 μM isobutyl-1-methylxanthine (IBMX) was added, and cells were incubated for an additional 48 h at 37 °C. The cells were washed twice with phosphate buffer, and then dissolved in 100 μL NaOH (1 N) containing 10% DMSO. Samples were incubated at 80 °C for 1 h and mixed to solubilize the melanin. The optical density of the mixed homogenate was measured at 490 nm. To measure the amount of melanin in each experiment, the total amount of melanin (100%) produced during the experimental period was considered as the control group, and the rate of inhibition in the treatment groups was calculated in proportion to this standard.

#### 3.6.4. Intracellular Tyrosinase Activity

Tyrosinase activity was determined as described by Li *et al.* [44] with slight modifications. B16F10 cells were plated at a density of 7 × 10^3^ cells/well in a 96-well plate. After 48 h of culture, cells were exposed to varying concentrations of *tairin* and *shima* EO (10, 20, and 50 μg/mL) or 500 μM kojic acid. After 1 h, 100 μM isobutyl-1-methylxanthine (IBMX) was added, and the cells were incubated for an additional 48 h at 37 °C. The cells then were washed with ice-cold phosphate buffer and lysed with phosphate buffer (pH 6.8) containing 1% Triton-X (90 μL/well). The plates were frozen at −80 °C for 30 min. After thawing and mixing, 10 μL of 1% l-DOPA was added to each well. Following incubation at 37 °C for 2 h, the absorbance was measured at 490 nm.

### 3.7. Statistical Analysis

Each experiment was performed in triplicate. All data are presented as the mean ± SE. Statistical analysis was done by one-way analysis of variance (ANOVA) complemented by the Duncan test. Differences were considered significant when the *p* value was less than 0.05.

## 4. Conclusions

This report is the first to demonstrate the anti-aging, anti-oxidant, and anti-melanogenesis activities of EOs from *tairin* and *shima* in cell culture. The present study reveals that EOs from *tairin* and *shima* have strong anti-oxidant activities against DPPH, ABTS, nitric oxide, singlet oxygen, and the hydroxyl radical, and inhibit xanthine oxidase. *Tairin* EO is a stronger inhibitor against a series of aging enzymes, such as collagenase, tyrosinase, hyaluronidase, and elastase, than s*hima* EO. Moreover, both *tairin* and *shima* EOs are anti-melanogenic.
